# Modified Gadonanotubes as a promising novel MRI contrasting agent

**DOI:** 10.1186/2008-2231-21-53

**Published:** 2013-07-01

**Authors:** Rouzbeh Jahanbakhsh, Fatemeh Atyabi, Saeed Shanehsazzadeh, Zahra Sobhani, Mohsen Adeli, Rassoul Dinarvand

**Affiliations:** 1 Department of Pharmaceutics, Faculty of Pharmacy, Tehran University of Medical Sciences, Tehran 14174, Iran; 2 Nanotechnology Research Centre, Faculty of Pharmacy, Tehran University of Medical Sciences, Tehran 14174, Iran; 3 Department of Biomedical Physics and Engineering, School of Medicine, Tehran University of Medical Sciences, Tehran, Iran; 4 Department of Chemistry, Sharif University of Technology, Tehran, Iran; 5 Department of Chemistry, Faculty of Science, Lorestan University, Khoramabad, Iran

**Keywords:** Carbon nanotubes, Contrast agent, MRI, Functionalization, Gadolinium, Pegylation

## Abstract

**Background and purpose of the study:**

Carbon nanotubes (CNTs) are emerging drug and imaging carrier systems which show significant versatility. One of the extraordinary characteristics of CNTs as Magnetic Resonance Imaging (MRI) contrasting agent is the extremely large proton relaxivities when loaded with gadolinium ion (Gd_n_^3+^) clusters.

**Methods:**

In this study equated Gd_n_^3+^ clusters were loaded in the sidewall defects of oxidized multiwalled (MW) CNTs. The amount of loaded gadolinium ion into the MWCNTs was quantified by inductively coupled plasma (ICP) method. To improve water solubility and biocompatibility of the system, the complexes were functionalized using diamine-terminated oligomeric poly (ethylene glycol) via a thermal reaction method.

**Results:**

Gd_n_^3+^ loaded PEGylated oxidized CNTs (Gd_n_^3+^@CNTs-PEG) is freely soluble in water and stable in phosphate buffer saline having particle size of about 200 nm. Transmission electron microscopy (TEM) images clearly showed formation of PEGylated CNTs. MRI analysis showed that the prepared solution represents 10% more signal intensity even in half concentration of Gd^3+^ in comparison with commerciality available contrasting agent Magnevist®. In addition hydrophilic layer of PEG at the surface of CNTs could prepare stealth nanoparticles to escape RES.

**Conclusion:**

It was shown that Gd_n_^3+^@CNTs-PEG was capable to accumulate in tumors through enhanced permeability and retention effect. Moreover this system has a potential for early detection of diseases or tumors at the initial stages.

## Introduction

Carbon nanotubes (CNTs) have unique physicochemical properties in biomedical and biological applications; hence have attracted attentions in different fields of nanotechnology [1-3]. Large specific surface area, efficient thermal and electrical conductivities, high mechanical strength, heat release in a radiofrequency field and capability of carrying therapeutics and imaging agents are some of these multifunctional features [4,5]. One of the extraordinary characteristics of CNTs loaded with gadolinium is their extremely large proton relaxivities which potentially could be used as magnetic resonance imaging (MRI) contrast agents (CA).

MRI is a powerful noninvasive imaging technique based on the differences between proton relaxation rates of water [6]. To enhance the contrast between different tissues and to detect disease states, using MRI CAs is inevitable [7,8]. Gadolinium (Gd^3+^) with seven unpaired electrons and large magnetic moment is a suitable agent for this purpose. Although the equated Gd^3+^ ion is toxic, the most contrast enhancements are based on Gd^3+^. Chelation or encapsulation of Gd^3+^, decreases the toxicity of this ion for medical applications [7,9]. One of the most commercially used CA is gadolinium-diethylene triamine penta acetic acid, (Gd^3+^-DTPA) commercially available as Magnevist®. Due to lack of specificity and sensitivity, this product is not very effective in early detection of the disease, so it has been classified as a traditional CAs [8].

Sitharaman et al. developed the first CNT-based contrast agent. They demonstrated that Gd@Ultra-short single-walled carbon nanotubes (gadonanotubes) drastically increase MRI efficacy compared to the traditional CAs [9]. However, the most challenging part of using CNTs in biological system is lack of solubility and hence its toxicity. Even though oxidation of CNTs improve their dispersibility, but it`s still not enough to call them as a suitable carriers. Wrapping biocompatible and biodegradable polyethylene glycol (PEG) onto the CNTs makes them soluble and helping them to escape reticuloendothelial system (RES) uptake. This modification causes longer blood circulation of CNTs and facilitates the passive targeting to the cancer cells through the enhanced permeability and retention (EPR) effect of tumor blood vessels [10-12]. Accordingly these particles can be applied for detection of tumors at the early stages.

In this work multi walled CNTs were functionalized by PEGylation and loaded with Gd_n_^3+^ enhance contrast effect of commercial Gd. T_1_/T_2_ measurements revealed that signal intensity of Gd_n_^3+^@CNTs-PEG was more than commercial Magnevist®.

## Methods and materials

### Oxidation of MWCNTs

**MWCNTs** (number of walls 3–15, outer diameter 5–20 nm, and length 1–10 μm) were purchased from Plasmachem (GmbH, Berlin, Germany). CNTs were oxidized according to the procedure reported before [13]. Briefly, 20 ml of sulfuric acid and nitric acid mixture (3:1 v/v) were added to 1 g of MWCNTs in a reaction flask and the mixture was sonicated for 30 min. Reaction medium was refluxed for 21 h at 120°C. The mixture was cooled and diluted with 1 L of distilled water, filtered, and washed with deionized water to adjust pH to ≈ 6. The product was dried by vacuum oven.

### Loading of GdCl_3_ (H_2_O)_6_ into the oxidized MWCNTs

100 mg of oxidized MWCNTs (O-MWCNTs) and 100 mg of GdCl_3_.6H_2_O (REacton®, 99.9%) were stirred together in 100 ml deionized water and sonicated in a bath sonicator for 60 min. The solution was left undisturbed overnight whereupon the Gd^3+^-loaded oxidized CNTs (Gd_n_^3+^@CNTs) flocculated from the solution. The supernatant was then decanted off. To remove any unabsorbed GdCl_3,_ remained sediment was dispersed in 25 ml of fresh deionized water with batch sonication and again, the Gd_n_^3+^@CNTs flocculated from solution was collected by decantation. This procedure was repeated 3 times. The final product was dried by vacuum oven.

### Functionalization of Gd_n_^3+^@CNTs with PEG_1500N_

28 mg of Gd_n_^3+^@CNTs was mixed with 474 mg Poly (ethylene glycol) bis (3-aminopropyl) terminated (M_n_~1,500, Aldrich) and the mixture was stirred at ≈ 120°C under nitrogen atmosphere for 6 days. Upon the addition of deionized water to the mixture, the suspension was placed in a membrane tube (molecular weight cutoff ~12000) for dialysis against fresh deionized water for 3 days to remove free PEG. Dialysis phases were also collected for the confirmation of absence of free Gd^3+^ ion by ICP. To removing large nanotube bundles the suspension was centrifuged three times at 13000 rpm for 15 min and the supernatant was freeze-dried.

### Determination of size and morphology

Dynamic light scattering (DLS) (Malvern Zetasizer ZS, Malvern UK) was used to determine the dynamic diameter and size distributions of Gd_n_^3+^@CNTs-PEG.

Transmission electron microscopy (TEM) and Thermal gravimetric analyses (TGA) (Shimadzu, Japan) was applied for characterization of preparation.

### ICP sample preparation

For ICP (Inductively Coupled Plasma) analysis, samples should digest with strong oxidizing agents like HNO3 or concentrated H2O2. As this harsh condition is not enough for digesting MWCNTs, in this study the nanotubes were first heated in oven at 650°C for 5 h. Fallowing cooling the sample, the solid residue was dissolved in the solution of HNO_3_(2%) and the Gd content was determined by ICP-Optical Emission Spectrometer (Varian 720-ES).

### In vitro T1/T2 measurement

The T1- and T2-weighted spin echo images at 1.5 Tesla (repetition time/echo time 250/16 msec and repetition time/echo time 4000/64 msec) were analyzed qualitatively. The signal intensities of vials with contrast medium in solution and contrast medium in cells with the corresponding Gd concentrations were visually compared.

For quantitative data analysis, the obtained MR images were transferred as digital imaging and communication in medicine (DICOM) images to a Dicom Works version 1.3.5 (DicomWorks, Lyon, France) [14,15]). For each concentration, three samplings and the maximum regions of interest were considered. Five concentrations of the carbon nanotubes (0.1818, 0.1, 0.05, 0.025 mM Gd or 0.1818, 0.1, 0.05, 0.025, 0.0125 mM/mL Gd) were prepared in sodium chloride 0.9%.

The imaging parameters were as follows: Standard Spin Echo, # of Echoes =1, TE=15 ms, TR=100, 200, 400, 600,1000, 2000 ms, Matrix=512*384, Slice Thickness=4 mm ,FOV=25 cm, NEX=3, Pixel Band width: 130 for T1 measurements and Standard Spin Echo, # of Echoes =4, TE=15/30/45/60 ms, TR=3000 ms, Matrix=512*384, Slice Thickness=4 mm, FOV=25 cm, NEX=3, Pixel Band width: 130 for T2 measurements.

T1 and T2 maps were calculated assuming mono exponential signal decay. T1 maps were calculated from four SE images with a fixed TE of 11 ms at 1.5T and variable TR values of 100, 200, 400, 600, 1000 and 2000 ms using a nonlinear function least-square curve fitting on a pixel-by-pixel basis. The signal intensity for each pixel as a function of time was expressed as follows (Equation 1) [16]:

$$\text{Signa}l_{\text{SE}1}\left(\text{TR},T_{1}\right)=S_{01}\left(1-e^{-\frac{\text{TR}}{T_{1}}}\right)$$

T2 maps were calculated accordingly from four SE images with a fixed TR of 3000 ms and TE values of 15, 30, 45, and 60 ms on the 1.5T MR scanner. The signal intensity for each pixel as a function of time was expressed as follows (Equation 2):

$$\begin{array}{l} \text{Signa}l_{\text{SE}4}\left(\text{TE},T_{2}\right)=S_{0}e^{-\frac{\text{TE}}{T_{2}}}\Rightarrow \text{ln}\left(\text{Signa}l_{\text{SE}4}\right)\\ \;\;\;\;\;=\text{ln}\left(S_{0}\right)-\frac{\text{TE}}{T_{2}} \end{array}$$

Care was taken to analyze only data points with signal intensities significantly above the noise level.

### Statistical analysis

One-way analysis of variance was used for comparison of the results. P values of 0.05 or less were considered as significant.

## Results and discussion

### Loading of Gd_n_^3+^ into the CNTs

In the presence study, MWCNTs were oxidized with harsh acid condition and then loaded with Gd_n_^3+^. Oxidizing occurred with the mixture of sulfuric and nitric acid (3:1). This procedure removes metal catalysts impurity and creates an open end termini in the structure and also sidewall defects that are stabilized by –COOH and –OH groups [12,17-19]. These hydrophilic holes are the very well place for accumulation of hydrophilic metal ions (e.g. Gd^3+^) on the surface or inside of the interior of a CNT [18,20]. Besides the –COOH group could be coupled to different chemical or biochemical groups [18-21].

The oxidized MWCNTs were loaded by soaking and sonicating them in double distilled water containing aqueous GdCl_3_. After sedimentation and dialysis to remove unloaded Gd^3+^ into the oxidized MWCNTs, Gd_n_^3+^@CNTs was functionalized with PEG. ICP analysis showed the Gd_n_^3+^ content of Gd_n_^3+^@CNTs and Gd_n_^3+^@CNTs-PEG to be 4.328% and 0.02% (w/w) respectively. The absence of free Gd^3+^ ion in the sample was confirmed by analysis the final dialysis medium through ICP, no detectable Gd^3+^ was shown.

### Solubilization and stabilization of Gd_n_^3+^@CNTs with PEG

**Figure 1:**
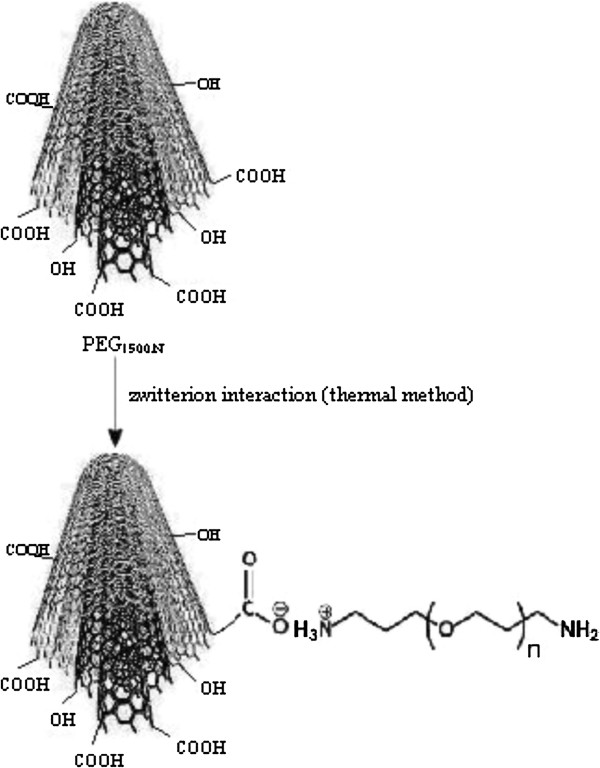
**Schematic diagram of functionalization of oxidized CNTs loaded with Gd**^**3+ **^**(Gd**_**n**_^**3+**^**@CNTs) with diamine-terminated PEG via zwitterion interactions.**

Carbon nanotubes have a rigid structure and presence in bundles, so they are essentially insoluble in any solvents. As a result, solubilization of CNTs via chemical functionalization has been attracted much recent attentions [1,5,10,17,18]. Among the possible hydrophilic polymers, with regard to biocompatibility, PEG is attractive for use with CNTs because of being nontoxic, properly stable and having a low immunogenicity [1,10,11,21]. Gd_n_^3+^@CNTs was functionalized with PEG_1500N_ (Gd_n_^3+^@CNTs-PEG). As reported by other researches, the attachment of diamine-terminated poly(ethylene glycol) with Gd_n_^3+^@CNTs were done via thermal reaction and zwitterion interaction between terminated amines of PEG and carboxylic groups of oxidized CNTs as shown in Figure 1[21].

**Figure 2:**
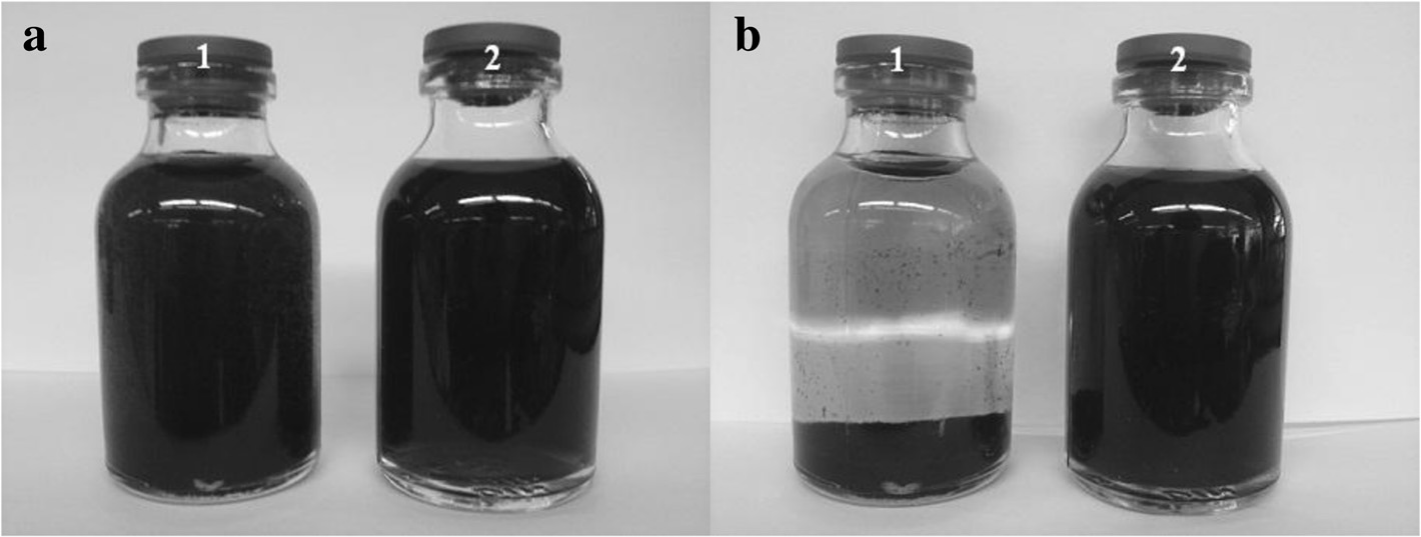
**Dispersion of Gd**_**n**_^**3+**^**@CNTs (1) and Gd**_**n**_^**3+**^**@CNTs-PEG (2) immediately after sonication in PBS (a) and 2 months later (b).**

As expected, the solution of the Gd_n_^3+^@CNTs-PEG was more stable than Gd_n_^3+^@CNTs in PBS. The Gd_n_^3+^@CNTs-PEG remained homogeneous over 2 months of observation time whereas in the Gd_n_^3+^@CNTs black precipitation appeared after a few days (Figure 2).

### Characterization of Gd_n_^3+^@CNTs-PEG

The particle size of Gd_n_^3+^@CNTs-PEG in water evaluated by Dynamic Light Scattering technique was about 200 nm with narrow poly dispersity index (PDI : 0.361). This particle size is appropriate for IV administration of solubilized gadonanotubes as a contrasting agent.

**Figure 3:**
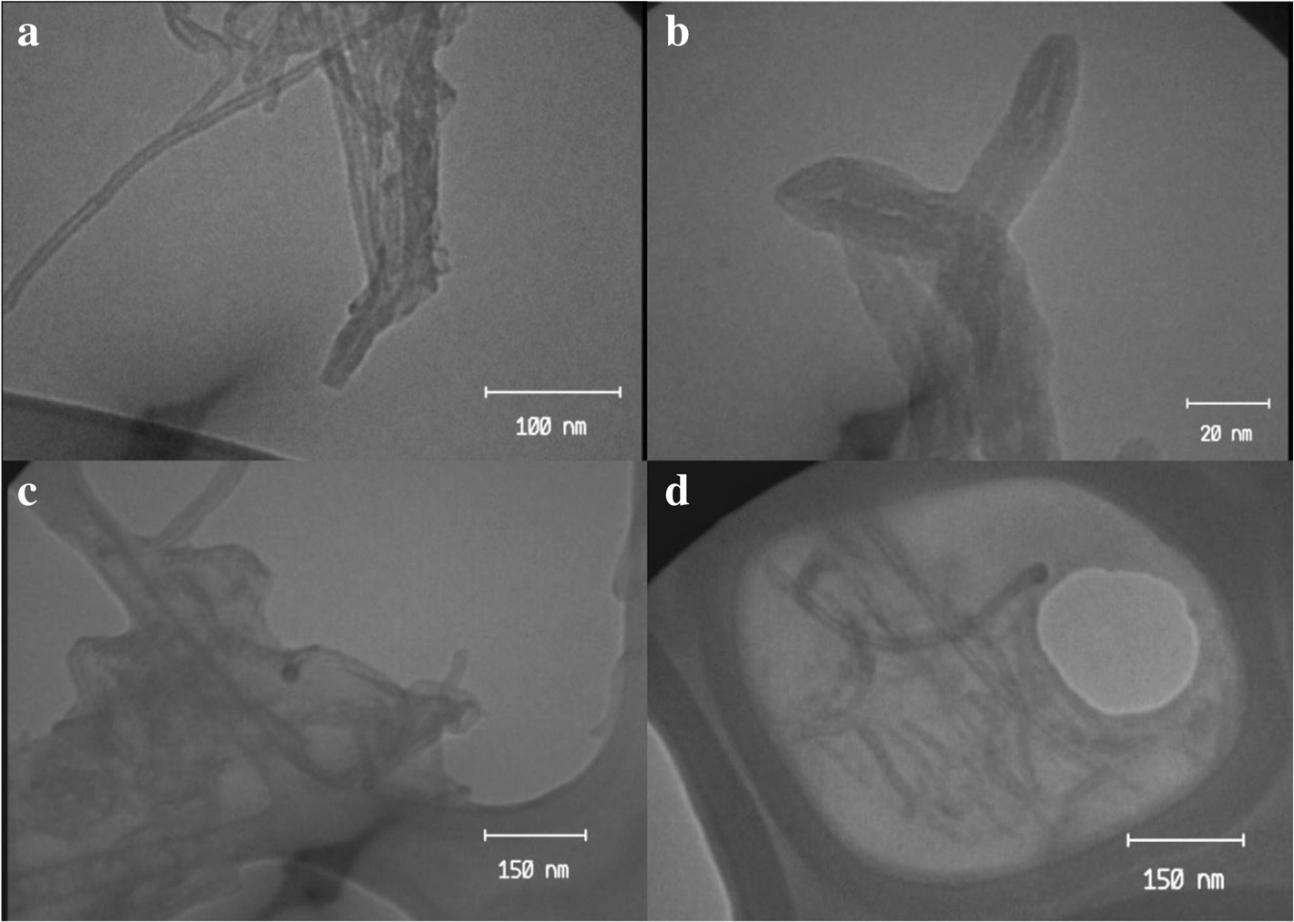
**TEM images of Gd**_**n**_^**3+**^**@CNTs (a-b) and Gd**_**n**_^**3+**^**@CNTs-PEG (c-d).**

Typical transmission electron microscopy (TEM) images of the functionalized MWCNTs loaded Gd^3+^ ions are shown in Figure 3. In the Gd_n_^3+^@CNTs-PEG image, wrapping PEG is can be clearly found around the nanotubes and the outer layer of polymer phase is discontinuous. Additionally nanotubes are dispersed either individually or in small bundles whereas in the image of Gd_n_^3+^@CNTs, tight bundles of nanotubes can be seen.

**Figure 4:**
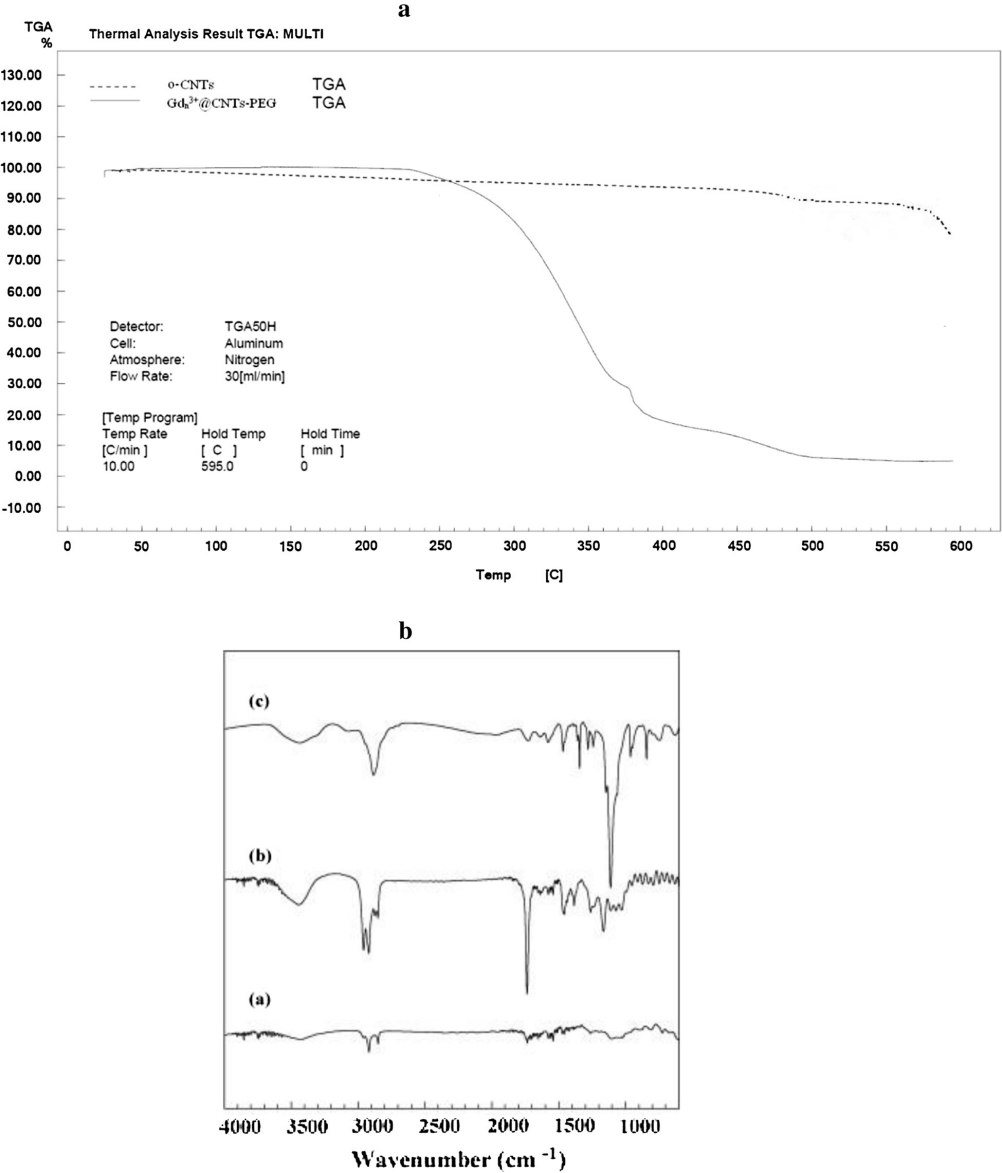
**a) TGA curves of oxidized CNTs (o-CNTs) and Gd**_**n**_^**3+**^**@CNTs-PEG, b) IR spectrum of modified CNT.**

Thermo gravimetric analysis (TGA) and IR spectroscopy was employed to determine either the tube is wrapped by polymer chains. Thermograms and IR spectrum of Gd_n_^3+^@CNTs-PEG and oxidized MWCNTs are shown in Figure 4. Wrapped PEG started to thermally degrade in the temperature range of 312°C. When the temperature reached to 450°C, PEG had essentially decomposed completely. According to the weight loss of PEG in Gd_n_^3+^@CNTs-PEG (about 95%), content of MWCNT in this compound is low. TEM images and ICP results also confirmed this low content of MWCNT in the Gd_n_^3+^@CNTs-PEG.

For oxidized MWCNTs, a weight loss was detected at 470°C, which can be attributed to the thermally unstable functional groups, e.g. –COOH and –OH on MWCNTs, formed during oxidation. These results indicate that PEG chains have successfully wrapped onto the MWCNT surfaces.

### T1/T2 measurement

T1/T2 measurements were performed in vitro, using magnetic resonance imaging apparatus. The analysis investigated that Gd_n_^3+^@CNTs-PEG solution in almost same and half concentration of Gd^3+^ compare to Magnevist® showed 29% and 9% more signal intensity respectively.

**Figure 5:**
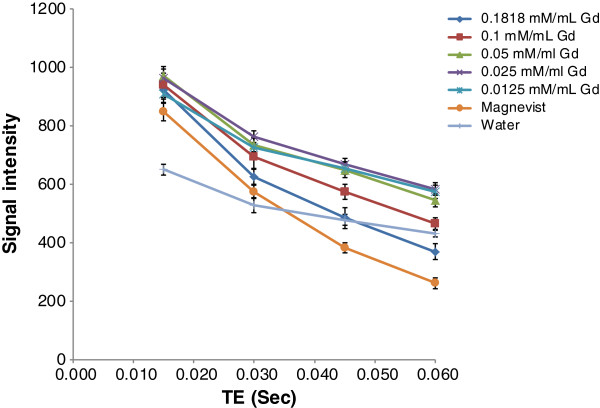
Signal changes based on echo time variation.

**Figure 6:**
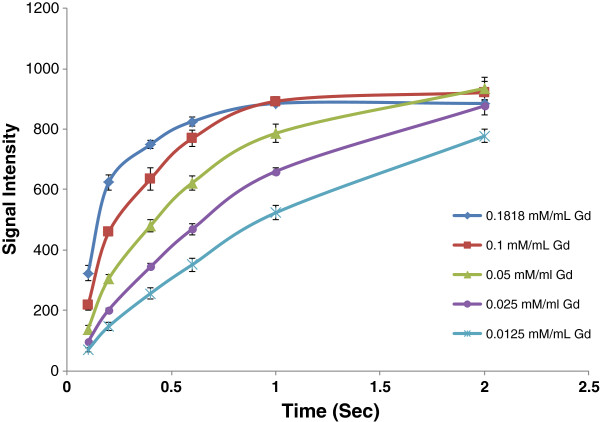
Signal changes based on repetition time variation.

The results of T1/T2 relaxation time (derived from equations 1 and 2) are shown in Tables 1 and 2 and Figures 5 and 6.

**Table 1:** T1 values (msec) derived from equations 1 and 2 for Gdn3+@CNTs-PEG with different Gd3+ concentration and Magnevist®

	**Concentration of Gd mM/mL**					**Magnevist®**	**Water**
TR (Sec)	**0.1818**	**0.1**	**0.05**	**0.025**	**0.0125**	**0.1818**	
T1	190.69	328.95	572.74	926.78	1385.23	405.35	3076.92
1/T1	0.005244	0.00304	0.001746	0.001079	0.0007219	0.002467	0.000325
R^^^2 value	**0.98**	**0.99**	**0.99**	**0.99**	**0.99**	**0.99**	**0.99**

**Table 2:** **T2 values (msec) derived from equations 1 and 2 for Gd**_**n**_^**3+**^**@CNTs-PEG with different Gd**^**3+ **^**concentration and Magnevist®**

	**Concentration of Gd mM/mL**					**Magnevist®**	
**TE (Sec)**	**0.1818**	**0.1**	**0.05**	**0.025**	**0.0125**	**0.1818**	**Water**
**1/T2**	19.95	15.29	12.36	10.90	9.85	6.42	8.80
**T2**	0.0501	0.0654	0.0809	0.0917	0.1015	0.1558	0.1136
R^^^2 value	0.99	0.99	0.99	0.98	0.97	0.99	96

**Figure 7:**
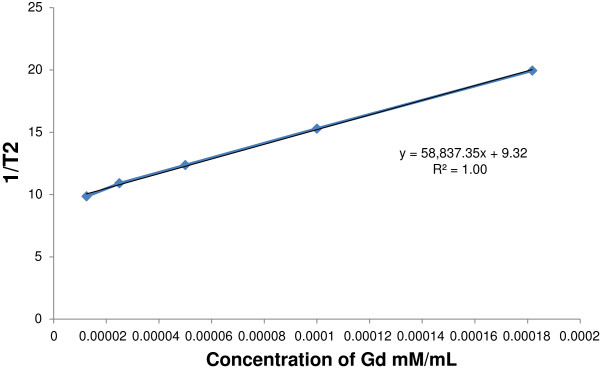
**1/T2 values at different Gd**^**3+ **^**concentrations.** The line slope represents the r_1_ value.

Gd_n_^3+^@CNTs-PEG clearly caused a significant decrease in both T1 and T2 relaxation time compared with Magnevist®. As shown in Table 1, the T1 values at the same concentration of Gd^3+^ in the Gd_n_^3+^@CNTs-PEG and Magnevist® were 190.7 msec and 405.4 msec, respectively. If we depicted the 1/T1 value at different Gd^3+^ concentration the r_1_ value will be obtained 26.6 (mMol^-1^.sec^-1^) as shown in Figure 7, while other studies show that the r_1_ value for Magnevist® was only 13.4 (mMol^-1^.sec^-1^) [22].

**Figure 8:**
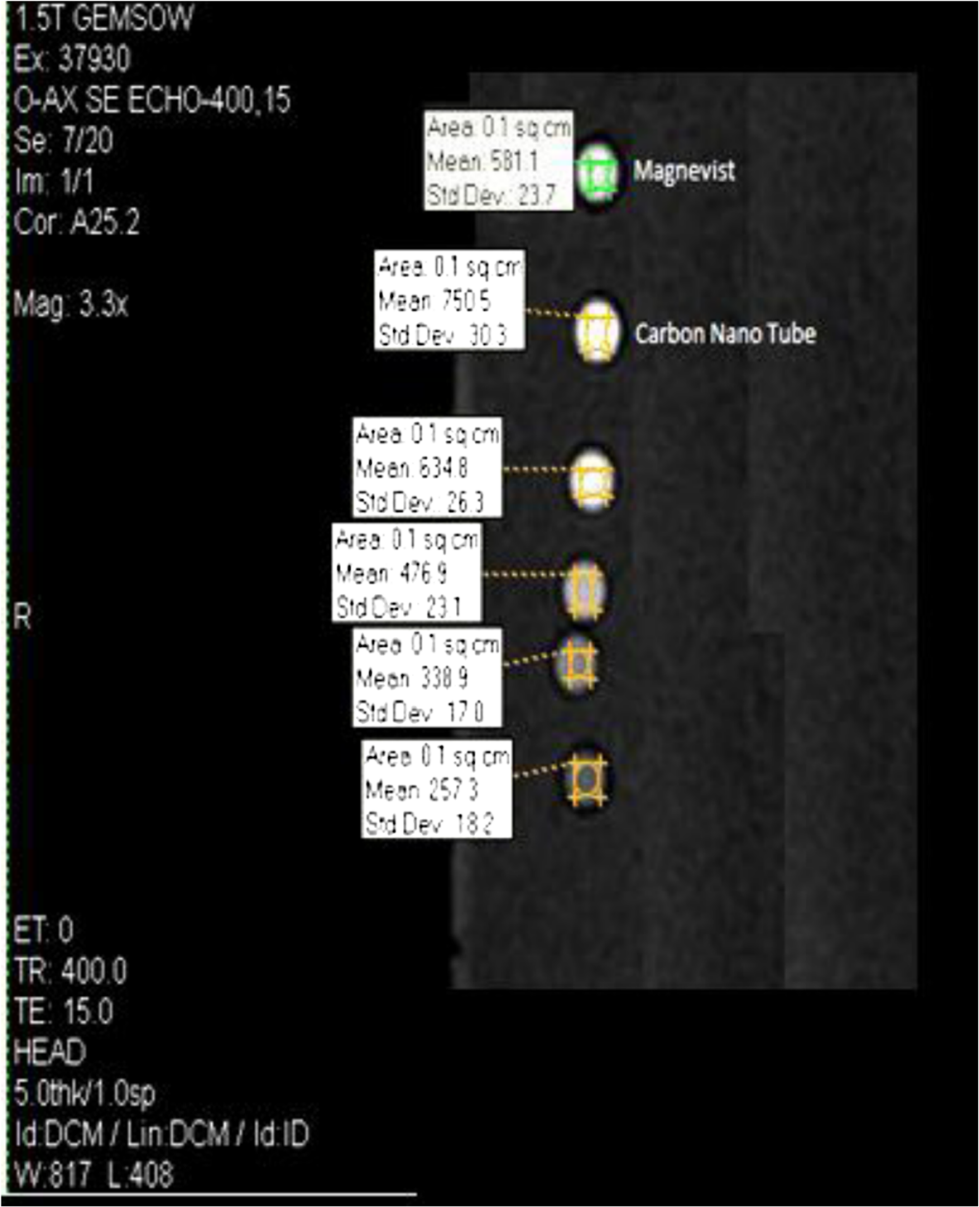
**The discrepancies among different concentrations of Gd**^**3+ **^**in the Gd**_**n**_^**3+**^**@CNTs-PEG and Magnevist® at T1 weighted image.** Five concentrations of the Gd_n_^3+^@CNTs-PEG (0.1818, 0.1, 0.05, 0.025 mMolarGd ) were sorted, respectively from top to bottom by diluting with sodium chloride 0.9%.

Table 2 shows the T2 values for Gd_n_^3+^@CNTs-PEG at different Gd^3+^ concentrations. The r_2_ value for Gd_n_^3+^@CNTs-PEG was 58.8 (mMol^-1^.sec^-1^) which was greater than Magnevist®. Data in tables and T1/T2 weighted images (Figure 8) showed that the signal increments of Gd_n_^3+^@CNTs-PEG were much higher even with half concentration of Gd^3+^ compare with the conventional contrast agent Magnevist®.

MR imaging of the samples (in test tubes) was performed using a 1.5T MR scanner (Signa, GE Medical Systems, Milwaukee, WI, USA) and a standard circularly polarized head coil (Clinical MR Solutions, Brookfield, WI, USA). All probes were placed in a water-containing plastic container (as shown in Figure 8) at room temperature (25°C) to avoid susceptibility artifacts from the surrounding air in the scans.

As shown in Figure 8 the signal intensity of Magnevist® and Gd_n_^3+^@CNTs-PEG at the same image condition, same protocol, same region of interest (ROI) area, and same Gd^3+^ concentration was 581.1 and 750.5, respectively. Therefore the signal intensity of Gd_n_^3+^@CNTs-PEG PEG was 29% and 9% more than Magnevist®, at equal or half of Gd^3+^ concentration, respectively.

## Conclusions

In order to increase proton relaxivity characteristics of gadolinium ion (Gd _n_^3+^ -ion) clusters, carbon nanotubes have been proven to be a good candidate. Addition of polyethylene glycol to this complex could improve the expected properties of the preparation as far as its solubility, stability and more over MRI contrasting ability of them. This could be the basis for further study to reach ideal goal which is detection of any abnormal tissues or tumors at the early stages.

## Competing interest

The authors declare that they have no competing interests regarding present work.

## Authors’ contributions

RJ conducted the experimental work and help in drafting the manuscript, FA conceived the study supervised the work and is the corresponding author of the work, SS performed the MRI experiments and ZS helped with the interpretation of the data analysis, MA helped with synthesis part of the work, RD reviewed and edited the manuscript. All authors read and approved the final manuscript.
